# Measuring Immunoglobulin Titers Among Healthcare Workers After Hepatitis B Vaccination: A Cross-Sectional Study in Kuwait

**DOI:** 10.7759/cureus.64910

**Published:** 2024-07-19

**Authors:** Sarah Alkhaldi, Hussah Aldousari, Shaikhah Alfaresi, Sarah Alqabandi, Walaa Khafagi, Marwa Sheha, Marwa Sanhoury, Alshaimaa Gomaa, Sahar Elshony, Farah Alenzi, Marwa Eltawansy

**Affiliations:** 1 Public Health, Kuwait Ministry of Health, Farwaniyah, KWT

**Keywords:** vaccination, anti-hbs, healthcare workers, hepatitis b vaccine, hepatitis b virus

## Abstract

Introduction

Hepatitis B virus (HBV) is a highly infectious disease affecting the liver, causing life-threatening acute and chronic hepatitis. It poses a significant global public health burden and is a major occupational risk for healthcare workers (HCWs) due to transmission through blood and blood products. Given their increased risk compared to the general population, vaccination is crucial in limiting the spread of HBV and protecting HCWs. This study aims to measure the anti-HBs titers of HCWs in the Farwaniyah Health District in Kuwait after completing three doses of the HBV vaccine.

Methods

This cross-sectional study was conducted in the Farwaniyah Health District of Kuwait between May and July 2023. We collected data from 556 participants from various departments, including physicians, nurses, and laboratory technicians, chosen through simple random sampling. Inclusion criteria included the completion of three doses of the HBV vaccine (Engerix-B recombinant vaccine, GlaxoSmithKline Biologicals, Brentford, UK). Demographic data were collected, and blood samples were tested for hepatitis B surface antigen antibody titers using fourth-generation enzyme-linked immunosorbent assay. Participants were categorized based on sex, age, specialty, and time since the last vaccine dose. The chi-square test and Fisher’s exact test assessed differences in categorical variables, with a p-value of <0.05 considered statistically significant.

Results

The study included 556 participants, with 304 (54.7%) women and 252 (45.3%) men. Participants were assigned into two age groups: 294 (52.9%) were 18 to 40 years old and the remainder (262) were over 40. Most participants were nursing staff (n=392; 70.5%), followed by physicians (n=110; 19.7%) and technicians (n=54; 9.7%). A high proportion (n=340; 61.2%) received their last vaccine dose within the last five years. Overall, 375 (67.4%) HCWs developed sufficient anti-HBs titers of ≥100 mIU/mL while 181 (32.6%) had levels below 100 mIU/mL. Age between 18 and 40 years and receiving the vaccine within the last five years were significantly associated with protective titer levels, while sex was not. Nurses had significantly higher immunity levels compared to doctors and technicians.

Conclusions

HCWs in the Farwaniyah area of Kuwait generally responded positively to the HBV vaccine. Younger HCWs and those who received the vaccine more recently were more likely to have a protective immune response. Nurses demonstrated higher rates of seroconversion compared to doctors and technicians. These results suggest that HBV vaccination programs should prioritize timely booster doses, especially for older HCWs and those vaccinated long ago. Monitoring antibody levels is crucial to ensure ongoing protection, particularly in high-risk groups such as nurses. Implementing these measures can enhance the effectiveness of HBV vaccination programs, reduce HBV incidence among HCWs, and contribute to a safer healthcare environment. Post-vaccination testing is essential to ensure the safety of all HCWs against HBV.

## Introduction

Hepatitis B virus (HBV) is a highly infectious disease that affects the liver, causing life-threatening acute and chronic hepatitis. This viral infection poses a significant global public health burden. According to the World Health Organization, 296 million people worldwide are chronically infected with HBV, with 60 million of them in the Eastern Mediterranean region [1]. In 2019, HBV was estimated to be responsible for 820,000 deaths worldwide [1].

HBV is transmitted parenterally through blood and blood products, making it a significant occupational risk for healthcare workers (HCWs). Studies have shown that HCWs injured by needles contaminated with both hepatitis B surface antigen (HBsAg) and hepatitis B e (HBeAg) antigen have a 37% to 62% risk of developing serologic evidence of HBV infection [2]. Another study reveals that approximately 66,000 HCWs acquire HBV infections annually due to occupation-associated sharps injuries, with an incidence rate of 5.9% [3].

Given the increased risk of HBV transmission among HCWs compared to the general population [4], vaccination plays a crucial role in limiting the spread of the disease and protecting HCWs. Completing three doses of the HBV vaccine has been shown to provide immunity against HBV infection [5]. The efficacy of the vaccine is measured by the levels of anti-HBs antibodies. The Centers for Disease Control and Prevention (CDC) reports that seroprotection against HBV is achieved with anti-HBs levels of ≥10 mIU/mL [6]. Additionally, the Hepatitis B Vaccination Greenbook states that an individual with a titer above 10 mIU/mL is considered protected; however, for healthcare and laboratory workers, levels above 100 mIU/mL are preferable [7]. This level ensures that further doses of the vaccine and additional titer testing are not required. Consequently, it is recommended that the HBV vaccine be enforced among HCWs and that antibody levels be measured to ensure adequate protection [8]. Our study aims to measure the anti-HBs titers of HCWs in the Farwaniyah Health District in Kuwait after three doses of the HBV vaccine, providing insight into the vaccine’s effectiveness in conferring immunity to HCWs.

## Materials and methods

Study design and population

This cross-sectional study was conducted in the Farwaniyah Health District of Kuwait between May and July 2023. Using the Andrew Fisher formula, with a 95% confidence level, a standard deviation of 0.5, and a 5% margin of error, the sample size representing the Farwaniyah health population was determined to be at least 385. We collected data from 556 participants from various departments, including physicians, nurses, and laboratory technicians, chosen through simple random sampling. The inclusion criteria for our study population were the completion of three doses of the hepatitis B vaccine (Engerix-B recombinant vaccine, GlaxoSmithKline Biologicals, Brentford, UK). Additional demographic data were collected from the participants’ records available in our public health department. After obtaining consent from the participants, blood samples were collected and sent to the microbiology laboratory of the public health department. The measurement of HBsAg antibody titers was performed using fourth-generation enzyme-linked immunosorbent assay.

Data analysis

Our study participants were categorized based on sex, age, specialty, and the time since receiving their last dose. These variables were plotted against the anti-HBs titers. We considered the cut-off level of 100 mIU/mL for HCWs as a positive response. Statistical analysis was conducted using IBM SPSS Statistics for Windows, Version 20.0 (Armonk, NY: IBM Corp.). The chi-square and Fisher’s exact tests were used to assess differences in the categorical variables. A p-value of <0.05 was considered statistically significant.

Ethical considerations

The study was submitted to and approved by the Standing Committee for Coordination of Health and Medical Research of the Kuwait Ministry of Health, as well as the Director of the Farwaniyah Health Area (approval no. 2023/2357). Consent was obtained from all participants prior to blood collection.

## Results

Our study included 556 participants who met the inclusion criteria. Among them, 304 (54.7%) were female and 252 (45.3%) were male (Table 1).

**Table 1:** Characteristics of study participants (N = 556)

Characteristics		Number	Frequency
Sex	Male	252	45.3%
	Female	304	54.7%
Age (years)	18-40	294	52.9%
	>40	262	48.1%
Specialty	Physician	110	19.7%
	Nurse	392	70.5%
	Technician	54	9.7%
Years since last dose	≤5 years	340	61.2%
	>5 years	216	38.8%
Total number		556	100%

Participants were divided into two age groups: 294 (52.9%) were aged 18 to 40, and the remainder (n=262; 48.1%) were over 40. The majority of participants were nursing staff (n=392; 70.5%), followed by physicians (n=110; 19.7%) and technicians (n=54; 9.7%). A high proportion (n=340; 61.2%) received their last dose of the vaccine within the last five years.

Overall, 375 HCWs (67.4%) developed sufficient anti-HBs titers of ≥100 mIU/mL while 181 (32.6%) had levels below 100 mIU/mL. After assessing four different categorical variables in relation to having protective anti-HBs titer levels, sex was the only category not significantly associated with the outcome (p = 0.103). Ages 18 to 40 years and receiving the vaccine within the last five years were both significant factors, with p = 0.007 and p = 0.00001, respectively. Additionally, immunity against HBV was significantly higher in nurses compared to doctors and technicians (p = 0.007; Table 2).

**Table 2:** Association of different categorical variables with HBsAg antibody titer concentrations *Statistically significant difference at p ≤ 0.05. **Fisher exact test is significant at p ≤ 0.05. HBsAg, hepatitis B surface antigen; NA, not applicable

Variables		HBsAg Antibody Titer Concentrations		X^2^	P-Value
		<100 mIU/mL, n (%)	≥100 mIU/mL, n (%)		
Total participants		181 (32.6%)	375 (67.4%)	NA	NA
Sex	Male	91 (36.1)	161 (63.9%)	2.656	0.103
	Female	90 (29.6)	214 (70.4%)		
Age (years)	18-40	79 (26.9%)	215 (73.1%)	9.178	0.002*
	>40	102 (38.9%)	160 (61.1%)		
Specialty	Physician	47 (42.7%)	63 (52.3%)	9.665	0.007*
	Nurse	112 (28.6%)	280 (71.4%)		
	Technician	22 (4.7%)	32 (59.3%)		
Time since last dose	≤5 years	0 (0%)	340 (100%)	NA	< 0.001**
	>5 years	181 (83.8%)	35 (16.2%)		

## Discussion

HBV is a recognized serious occupational risk among HCWs worldwide. Although Kuwait has a comprehensive HBV vaccination program for all citizens, including HCWs, assessing the vaccine’s immunogenicity is crucial to ensure an adequate protective response for those at increased risk of exposure. Our study assessed 556 HCWs in the Farwaniyah Health District, aged 18 to 70 years, and measured anti-HBs antibody levels. We considered levels above 100 mIU/mL to be protective.

The overall seroprotection rate was 67.4%, comparable to findings from studies in Bulgaria, Sri Lanka, and India, which showed 62.7%, 66.2%, and 76.5%, respectively [9-11]. According to our study, 32.2% of participants had antibody titers below 100 mIU/mL even after receiving three vaccine doses. This necessitates providing an additional dose of the vaccine for those with antibody levels from 10 to 100 mIU/mL or repeating the vaccine course for non-responders (levels below 10 mIU/mL), according to UK guidelines [7].

Our study found a statistically significant difference between age groups below and over 40 years, with the older group showing lower titer levels. This finding is consistent with a systematic review by Yang et al. in 2016, which found lower vaccine response rates in adults aged 40 years and older [12]. This difference may be due to older participants receiving their vaccine earlier, affecting titer levels over time, as shown in other studies [10,13]. Our study also demonstrated that titer levels decline over time since receiving the last vaccine dose. Sex did not appear to affect vaccine immunogenicity, consistent with other published studies [10,14]. Notably, nurses had significantly higher titer levels than doctors and technicians, which might be due to their more stringent adherence to vaccination schedules and boosters, given their higher exposure to needle-stick injuries [15].

Our study faced several limitations. We did not obtain certain factors, such as vaccination schedules and receipt of booster doses, which should be explored concerning the outcome. Participants with anti-HBs titers below 100 mIU/mL should be further tested to determine if they are hypo-responders (i.e., they have antibody levels between 10-100 mIU/mL) or non-responders (i.e., they have antibody levels below 10 mIU/mL) and managed accordingly. Furthermore, the study does not take into account whether the participants have been vaccinated within one to two months before obtaining the titer levels. This information is crucial to determine the next step of action.

We recommend implementing a public health program in Kuwait to screen HCWs for immunity post-immunization. Once HCWs are documented to be hyper-responsive, further primary doses and retesting are unnecessary [8], which could reduce the economic burden on the Ministry of Health.

## Conclusions

HCWs in the Farwaniyah area of Kuwait who were vaccinated against HBV generally responded positively to the vaccine. Younger HCWs and those who received the vaccine more recently were more likely to exhibit a protective immune response. Among the various healthcare professions, nurses demonstrated higher seroconversion rates than doctors and technicians. The results imply that HBV vaccination programs should prioritize timely booster doses, especially for older HCWs and those who received the vaccine longer ago. Additionally, there should be an emphasis on monitoring antibody levels to ensure ongoing protection, particularly in high-risk groups such as nurses. These measures can enhance the overall effectiveness of HBV vaccination programs, reduce the incidence of HBV among HCWs, and ultimately contribute to a safer healthcare environment. Post-vaccination testing is essential to ensure the safety of all HCWs against HBV, highlighting the need for ongoing monitoring and booster vaccinations where necessary.

## References

[1] (2023). Hepatitis B. https://www.who.int/news-room/fact-sheets/detail/hepatitis-b.

[2] Mauss S, Berg T, Rockstroh J, Sarrazin C, Wedemeyer H (2015). Hepatology. 6th Edition. https://www.saudedireta.com.br/catinc/tools/e_books/Hepatology2015.pdf.

[3] Prüss-Ustün A, Rapiti E, Hutin Y (2005). Estimation of the global burden of disease attributable to contaminated sharps injuries among health-care workers. Am J Ind Med.

[4] Dannetun E, Tegnell A, Torner A, Giesecke J (2006). Coverage of hepatitis B vaccination in Swedish healthcare workers. J Hosp Infect.

[5] Puvačič S, Ravlija J, Puvačič Z, Curič I (2005). Long term protection after hepatitis B vaccination. Bosn J Basic Med Sci.

[6] (2023). Hepatitis B vaccine. https://www.cdc.gov/vaccinesafety/vaccines/hepatitis-b-vaccine.html.

[7] (2023). Hepatitis B: the green book, chapter 18. https://www.gov.uk/government/publications/hepatitis-b-the-green-book-chapter-18.

[8] (2023). Aide-memoire for a strategy to protect health workers from infection with bloodborne viruses. World Health Organization. https://www.who.int/publications/i/item/WHO-BCT-03.11.

[9] Ivanova L, Kyoseva M, Metodiev K, Stojkova J (2013). Serologic hepatitis B virus immunity in health care workers. Eur J Inflamm.

[10] Chathuranga LS, Noordeen F, Abeykoon AM (2013). Immune response to hepatitis B vaccine in a group of health care workers in Sri Lanka. Int J Infect Dis.

[11] Basireddy P, Avileli S, Beldono N, Gundela SL (2018). Evaluation of immune response to hepatitis B vaccine in healthcare workers at a tertiary care hospital. Indian J Med Microbiol.

[12] Yang S, Tian G, Cui Y (2016). Factors influencing immunologic response to hepatitis B vaccine in adults. Sci Rep.

[13] Ocan M, Acheng F, Otike C, Beinomugisha J, Katete D, Obua C (2022). Antibody levels and protection after Hepatitis B vaccine in adult vaccinated healthcare workers in northern Uganda. PLoS One.

[14] Perera J, Perera B, Gamage S (2002). Seroconversion after hepatitis B vaccination in healthy young adults, and the effect of a booster dose. Ceylon Med J.

[15] Jayaprada R, Vineela K, Ramakrishna N, Yamini S, Bhargav K (2022). A study of needle-stick injury incidence amongst healthcare workers and its root cause analysis in a tertiary care teaching hospital. J Clin Sci Res.

